# Estimating Binding Energies of π-Stacked Aromatic Dimers Using Force Field-Driven Molecular Dynamics

**DOI:** 10.3390/ijms25115783

**Published:** 2024-05-26

**Authors:** Daniel Doveiko, Karina Kubiak-Ossowska, Yu Chen

**Affiliations:** 1 Photophysics Group, Department of Physics, University of Strathclyde, Scottish Universities Physics Alliance, Glasgow G4 0NG, UK; daniel.doveiko.2018@uni.strath.ac.uk; 2 Department of Physics/ARCHIE-WeSt, University of Strathclyde, Glasgow G4 0NG, UK

**Keywords:** π–π stacking, polycyclic aromatic hydrocarbons, PAH, molecular dynamics, MD, steered molecular dynamics, SMD, DFT, density functional theory

## Abstract

π–π stacking are omnipresent interactions, crucial in many areas of chemistry, and often studied using quantum chemical methods. Here, we report a simple and computationally efficient method of estimating the binding energies of stacked polycyclic aromatic hydrocarbons based on steered molecular dynamics. This method leverages the force field parameters for accurate calculation. The presented results show good agreement with those obtained through DFT at the ωB97X-D3/cc-pVQZ level of theory. It is demonstrated that this force field-driven SMD method can be applied to other aromatic molecules, allowing insight into the complexity of the stacking interactions and, more importantly, reporting π–π stacking energy values with reasonable precision.

## 1. Introduction

π–π stacking (pi-stacking) are reversible and noncovalent interactions between aromatic rings that contain π-orbitals [1,2], which are often crucial in many areas of chemistry, biology, material science, and others [3]. Although these interactions are relatively weak individually, their effect can be significant, as the formation of oligomers driven by π–π stacking can affect the properties of those oligomers [4]. 

The simplest prototype of π–π stacking, extensively studied using both experimental [5,6] and computational methods [7,8] with the hope of obtaining a clear picture of the directionality and strength of π–π stacking, is the benzene dimer. The benzene dimerization occurs due to the quadrupolar moment introduced by the delocalized π-electrons, resulting in electrostatic interactions that compete with the dispersion forces, with the most favorable configurations of dimers being parallel or perpendicular [9]. Due to the low binding energy (2–3 kcal/mol) of gaseous benzene dimer, modeling and experimental measurements of such constructs are challenging, often requiring complex and time-consuming quantum calculations involving large datasets or low temperatures to reduce the kinetic energy of the aromatic molecules [10]. Furthermore, the description of such interactions is often misleading, as in the case of direct stacking, sometimes referred to as “sandwich”, the resulting interactions are strongly repulsive; hence, more commonly observed are staggered interactions, where the parallel aromatic rings are horizontally displaced [11]. The repulsive nature of the “sandwich” configuration is caused by the electrostatic repulsion between negatively charged carbons and positively charged hydrogens placed parallel on top of each other, resulting in a strong repulsion; hence, such configurations are particularly rare [12]. In the usually observed staggered π–π stacking, where the rings are offset horizontally, the negatively charged carbon is placed on top of hydrogen bearing a positive charge, resulting in electrostatic attraction, which is then balanced via attractive van der Waals (vdW) interactions (attractive dispersion) and Pauli repulsion [13]. 

Considering all of the above, a question arises: How can force field-driven molecular dynamics (MD) simulations represent π–π stacking, which is based on quantum mechanical interactions? To answer this question, it is vital to understand how the potential energy function is defined in molecular dynamics simulations. In general, the force field (FF) terms contribute to the potential energy as follows:

(1) $$U_{bonds}>U_{angles}>U_{dihedrals}>U_{impropers}>U_{non-bonded}$$

Typically, in systems near equilibrium, the strongest contribution to the total potential energy function comes from the bonded interactions, while the non-bonded electrostatic Coulomb and vdW interactions have minor contributions. The π–π stacking comes from the $U_{non-bonded}$; hence, the bonded interactions will not be discussed further herein.

To understand how the inclusion of π–π stacking is achieved, it is crucial to understand what properties are described and included in van der Waals interactions. vdW interactions are caused by correlations in the fluctuating polarizations of nearby particles (a consequence of quantum dynamics [14]) and consist of four components [15]: (1) attractive or repulsive electrostatic interactions, referred to as Keesom interactions or Keesom forces [16]; (2) polarization effects, which are the attractive interactions between a permanent multipole on one molecule and an induced multipole on another, referred to as the Debye force [17]; (3) London dispersion forces, which are attractive interactions between any pair of molecules, including non-polar atoms, arising from the interactions of instantaneous multipoles [18]; and (4) a repulsive component resulting from the Pauli exclusion principle [19,20].

Although often overlooked due to its low energy compared with other types of interactions, vdW forces represented by certain parameters, as implemented in the MD force field, are instrumental when performing molecular simulations. Upon closer inspection of Table 1, one can see that each atom type has multiple parameters with marginally different ε and radii, each of them representing atoms from different chemical groups, e.g., in CHARMM FF atoms, CA describes sp^2^ carbons in the aromatic rings [21], so the ε and radius are unique to this specific type of carbon hybridization in this unique configuration. The same applies to all other atoms, such as hydrogens, oxygens, etc., and indirectly reflects the electronic structure of the atom.

It is worth mentioning, that a simple, protein-only FF consists of over ten types of hydrogen and nitrogen atoms and over twenty carbon atoms [22]. Due to those small and therefore frequently overlooked differences in ε and radii, force field-driven MD simulations, as we will show below, allow the reproduction of quantum effects, such as π–π stacking. Usually, such interactions are studied by implementing complex quantum chemical calculations, which typically tend to be very computationally expensive and complicated; therefore, the usage of a simpler, cheaper, and faster but still accurate method would have a massive potential for speeding up the research progress.

**Figure 1:**
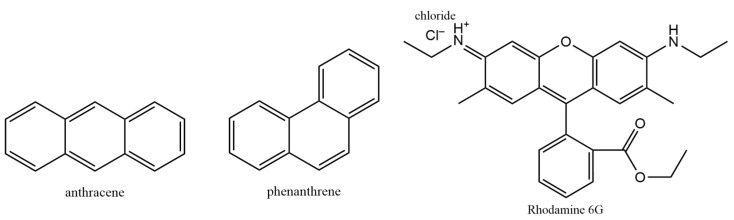
Chemical structures of the molecules utilized for the simulations.

To address this issue, we report a molecular dynamic/steered molecular dynamics (SMD)-based approach that allows us to gain insight and assess the precise values of binding energies previously achieved using computational quantum chemistry. To elucidate the details of π–π stacking and allow for the easier measurement of binding energies in such stacked structures, we performed simulations on well-studied polycyclic aromatic hydrocarbon (PAH, Figure 1) H-type dimers of phenanthrene and anthracene, each containing fourteen π-electrons, and on rhodamine 6G (R6G) H-type dimers, containing twelve π-electrons. Moreover, to reinforce the reliability of the force field-driven SMD approach, we conducted density functional theory (DFT) calculations at the ωB97X-D3/cc-pVQZ level of theory [24,25,26]. Our findings indicate comparable trends to those reported through SMD, affirming the consistency and accuracy of our methodology. This convergence of results underscores the fidelity of the CHARMM 36 parametrization. Therefore, we believe that the combined MD/SMD method presented herein can be used as a preliminary tool to quickly and accurately estimate the interaction energies between two PAH molecules using the force field-based approach. Nonetheless, the method presented here is applicable as a substitute for quantum chemical calculations, specifically in the estimation of binding energies, which are directly related to the force field parameters. However, for tasks necessitating frequency calculations, geometry optimization, charge distribution, etc., quantum chemical methods remain irreplaceable.

## 2. Results and Discussion

In this work, we performed MD simulations of free diffusion for benzene, anthracene, phenanthrene, and R6G molecules in water. For all molecules, spontaneous dimerization and dissociation were easily observed using both visual analysis (VMD [27]) and center of mass (COM) distance measurements between the two involved molecules. This spontaneous dimerization/dissociation process suggests that the energetical barriers are relatively low, and it is possible to cross them several times in 300 K and in a 100 ns time scale. In the next paragraphs, we concentrate on calculating those energies.

### 2.1. Force Field-Driven MD Simulations

**Figure 2:**
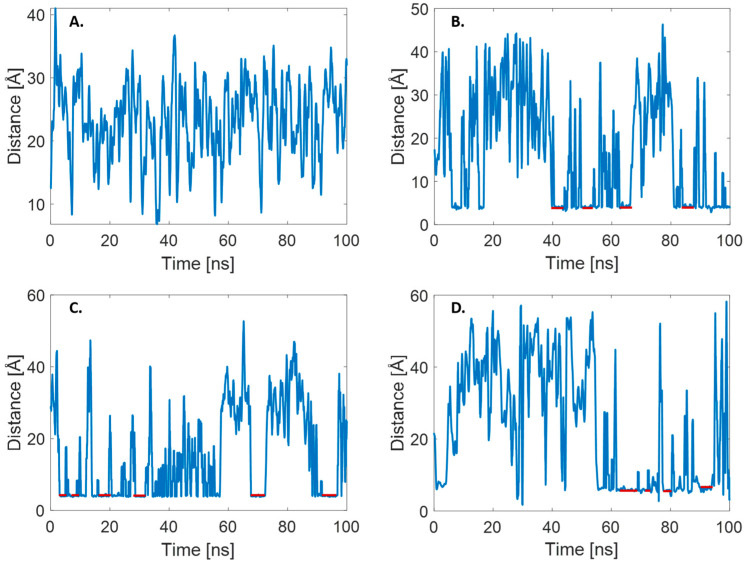
COM distance plots observed in the MD simulations of two molecules in an aqueous environment. (**A**) Benzene; (**B**) anthracene; (**C**) phenanthrene; (**D**) R6G. Red lines mark periods where stable dimers are formed due to the stacking interactions.

As mentioned previously, due to the low energy barriers and low binding energy, the benzene dimerization/dissociation process is very fast and labile and therefore incredibly difficult to study via MD, which is the case in the approach described herein as well. The lack of stable dimerization in benzene molecules can be identified as a lack of periods when the distance between two entities remains unchanged for a prolonged time, as shown in Figure 2A. In the case of two anthracene molecules shown in Figure 2B, stable dimers were constantly forming and dissociating, due to the higher binding energy and slower diffusion compared to benzene. Furthermore, the entire process in the case of anthracene was slower than that in the case of benzene, making the investigation via MD possible. The formation of dimers, due to π–π stacking, can be clearly identified from the prolonged periods of stable COM distance (as indicated in red on Figure 2B). The same features were observed in the case of phenanthrene (Figure 2C) and R6G (Figure 2D). Since the spontaneous creation of stable dimers was observed for all considered molecules except benzene, SMD was carried for anthracene, phenanthrene, and R6G only. Furthermore, our observations indicate that the only stable aggregates formed during the simulations were π-stacked dimers. T-shaped structures were either not observed, or if they were, they were highly unstable, as exemplified by the case of the benzene dimer.

The assessment of dimerization energy is conducted through SMD simulations. Analogous to traditional MD, SMD simulations leverage small variations in vdW parameters to model and precisely quantify certain quantum phenomena. This approach obviates the necessity for highly resource-intensive quantum chemical simulations, specifically pertaining to π–π stacking interactions. As the π–π stacking interactions are relatively weak (e.g., benzene dimer binding energy is on the order of 2–3 kcal/mol [10]) and thus difficult to measure, any molecular disturbance during the SMD trajectory must be minimal. This is achieved by switching off the constant temperature control, fixing one of the dimer components, and slowly pulling the other one with a constant velocity. Such an approach allows the reduction of noise in both force and displacement plots without losing any information, therefore resulting in more precise energy calculations of such subtle interactions.

**Figure 3:**
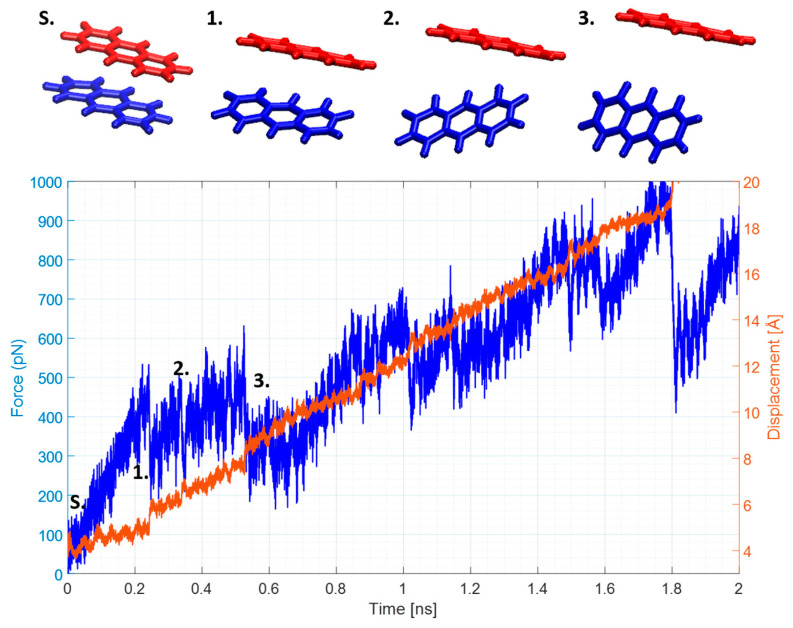
Anthracene SMD plots. Structure S represents the starting structures, while structures denoted as 1–3 represent the various stages of dimer dissociation in the SMD trajectory. The blue curve represents the force in pN, while the orange one represents the displacement in Å from the original position.

As vdW forces are short-range, any interaction between two molecules whose COMs are separated by 10 Å or over cannot be confidently identified as a stacking interaction and hence were not considered when calculating the binding energies of the stacked dimers. Any sudden increases in force values and displacement past that mark were caused mainly by the collisions with water molecules present in the system. As expected, forced by SMD simulations, H-type dimer dissociation was a multi-step process in all cases, and it reflected the fact that all of the molecules contained three aromatic rings that can participate in π–π stacking. By looking at the force and displacement curves and comparing them with the visual analysis of the trajectories, the binding energies Δ*E* can be estimated using the following relation, which is a re-expression of the potential energy of a spring (described in the Materials and Methods section):

(2) $$\Delta E=\left(F_{0}+\frac{dF}{2}\right)\left(\frac{dF}{k}\right)$$

where *F*_0_ is the force at the end of the transition, *dF* is the force change during the transition, and *k* is the spring constant equal to 278 pN/Å. This method has been successfully used for estimating the desorption energies of proteins [28,29,30]. The multistep anthracene dimer dissociation and corresponding SMD results are shown in Figure 3. 

An analogous type of analysis was performed for the phenanthrene and R6G dimers; force and displacement plots are shown in Figures S1 and S2. Although the details of all calculations are not presented for the clarity of the manuscript, the results are summarized in Table 2, which contains the averaged Δ*E*_SMD_ from the four independent SMD simulations, each starting from a different point in the force field-driven MD trajectory (details given in Materials and Methods section below). As expected, the values for anthracene and phenanthrene binding energies were virtually identical (10.23 ± 1.36 kcal/mol vs. 10.34 ± 2.24 kcal/mol)—both molecules had identical chemical composition (C_14_H_10_), and both had fourteen π-electrons, with the only difference being the spatially offset aromatic ring. Only the pulled molecule was dynamic during SMD simulations, while the water molecules remained stationary and vibrated in place. Therefore, any observed collisions or interactions occurred as a result of a collision between a pulled PAH molecule and a water molecule. This created a cavity around the PAH dimer (Figure S3 and Video S1), resulting in a simulation where explicit water molecules were present, but the SMD part seemed to behave in a gas-like phase. We address this phenomenon in detail in the next section.

Furthermore, it is important to note that the reduction in dimer binding energy for the case of R6G (Table 2) may potentially result from the electrostatic repulsion of ionic R6G molecules. However, precise estimation of this factor was not feasible, due to the nature of SMD, which reports the total force acting on the pulled atoms without differentiation. It is noteworthy that in our simulations, Cl ions used to neutralize the charge of R6G were allowed to diffuse freely. Contrary to expectations regarding electrostatic interactions and in line with previous reports on R6G dimerization [31,32], these ions did not facilitate the rhodamine dimerization. The results above suggest that, in this particular case, vdW interactions predominated over electrostatic repulsion. However, as demonstrated in the subsequent section, the dimerization was driven by a cooperative effect between vdW interactions and the environment, suggesting that the solvent played an essential role in forming stable dimers.

It is important to note that due to the current limitations of available models and force field parameters, the estimation of binding energies of T-shaped structures, which are known to be almost isoenergetic to the π-stacked dimers, or the correction of the R6G dimer binding energy arising from ion–ion interactions could not be achieved using the methods described herein. Furthermore, it is essential to recognize that the presented approach should not be a complete replacement for more precise quantum chemical calculations. Instead, it should be regarded as a guide to direct researchers towards the correct direction when estimating binding energies.

### 2.2. DFT Calculations

To gain deeper insights into the accuracy of the obtained Δ*E*_SMD_ values, we performed DFT calculations on the dimer structures, as reported by MD simulations, in solute without prior geometry optimization. All results were obtained using the ωB97X-D3 functional [24,25] utilizing Grimme’s dispersion correction with the cc-pVQZ basis set [26]. As expected, the obtained Δ*E*_SMD_ values showed reasonably good agreement with those obtained using DFT with implicit solvation (${\Delta E}_{DFT}^{Solvent}$), namely the energies of 9.41 ± 0.64 kcal/mol for anthracene and 11.04 ± 0.36 kcal/mol for phenanthrene. Furthermore, the Δ*E*_SMD_ value of 8.52 ± 2.80 kcal/mol for the R6G dimer was in agreement with the ${\Delta E}_{DFT}^{Solvent}$ of 13.45 ± 3.18 kcal/mol, further validating the CHARM36 FF and suggesting the efficacy of our SMD approach in estimating the binding energies of π-stacked dimers, extending beyond simple PAH. As already mentioned, during SMD simulations, a cavity was created around the molecule of interest, potentially implying a gas-like phase for the SMD portion. However, the results listed in Table 2 indicate that comparing Δ*E*_SMD_ and ${\Delta E}_{DFT}^{Gas}$ is not a valid approach, as the energy values in these two cases differed significantly. Moreover the ${\Delta E}_{DFT}^{Solvent}$ values were on average 2 kcal/mol higher for PAH compared to ${\Delta E}_{DFT}^{Gas}$, suggesting that the dimers were stabilized by water. The results show that the solvent plays a critical role in forming stable R6G dimers, as the obtained ${\Delta E}_{DFT}^{Gas}$ was −19.06 ± 2.16 kcal/mol, implying that dimer formation is energetically unfavorable in the gas phase. In the solvent, the binding energy ${\Delta E}_{DFT}^{Solvent}$ increased to 13.45 ± 3.18 kcal/mol, which aligns within the uncertainty Δ*E*_SMD_ equal to 8.52 ± 2.80 kcal/mol. This suggests that the stable dimer formation can occur only in solution arising from the cooperative effect between the vdW forces and the solvent. This indicates that the physicochemical properties of the studied system may vary significantly between environments; thus, binding energies in the solvent and gas phase should not be identical. Consequently, direct comparisons should be approached with caution. It should be noted that the presented DFT results are preliminary and should be used only to compare trends, as they are based on unoptimized structures and do not account for vibrational effects and basis set superposition error, which would have a notable effect on the final binding energy values.

It is important to note that the accuracy of the result strongly correlates with the functional being used. The double-hybrid functionals, such as PWPB95 or B2PLYP, are the pinnacle of chemical accuracy, although they are the most computationally demanding within the DFT family, while the outdated traditional Hartree–Fock methods can be referred to as Hartree “hell” in terms of their precision, compared to modern functionals [33,34]. Furthermore, the size of the basis set and the software selection may affect the computational speed and accuracy of the results [33,35,36]. It is worth mentioning that the relatively large size of the R6G dimer might present a challenge for quantum chemical calculations at a higher level of theory like those involving larger basis sets combined with coupled-cluster theory. While such calculations are considered the gold standard for single-point calculations, they are often not feasible within a comparable computational timeframe, compared to those obtained via our proposed force field-based approach. However, our findings demonstrate that the Δ*E*_SMD_ values for anthracene, phenanthrene, and R6G π-stacked dimers agree with the ${\Delta E}_{DFT}^{Solvent}$ values. These alignments suggest that our presented SMD method holds promise for accurately estimating the binding energies of other π-stacked dimers as well.

### 2.3. Summary

In conclusion, we have presented a precise, straightforward, and computationally efficient method for assessing binding energies in π–π stacked PAH H-type dimers, focusing on phenanthrene and anthracene, and successfully extended its application to an H-type R6G dimer. The obtained Δ*E*_SMD_ values are in agreement with the performed DFT calculations, further emphasizing the quality of the CHARMM36 parameters. Thus, our study serves as additional validation of the accuracy of these parameters. We demonstrated that by switching off the temperature control in SMD trajectories and due to the inclusion of quantum properties of the atoms in the vdW parameters, it is possible to measure the binding energies of π-stacked PAH dimers of various sizes quickly and reliably. This further suggests that the proposed method can be applied to various PAH dimers and its variants that are too large to be effectively targeted via an expensive quantum approach. 

Despite the apparent agreement between the results obtained from different methods, it is necessary to proceed with caution when comparing the exact values because quantum and force field-driven calculations are usually performed in different environments, namely gas phase vs. solvated molecules.

## 3. Materials and Methods

Aromatic carbon structures were built using the Nanomaterial Modeler extension in CHARMM-GUI (Lehigh University, Bethlehem, PA, USA) [37,38], while the initial R6G structure was taken from our previous work [39]. All simulations were run using the NAMD3 CUDA (University of Illinois Urbana–Champaign, Champaign, IL, USA) version [40,41] and CHARMM36 FF [22]. The minimization of the system was performed in two steps: (1) water only (1000 minimization steps and 100 ps equilibration in T = 300 K) and (2) the entire system (10,000 minimization steps followed by 30 ps of heating to 300 K and 270 ps of thermalization with a 1 fs time step). In the MD production stage, the integration step was 1 fs to minimize potential errors, while the total length of the trajectory was 100 ns to ensure the molecules had enough time for spontaneous dimerization and dissociation. 

Particle mesh Ewald (PME) was used for the electrostatic interactions, and vdW cut-off was set to 12 Å. For the water, the TIP3P [23] model was employed while the internal water molecule vibrations were constrained. The anisotropic cell fluctuations ensured that the desired pressure of 1 atm at 300 K was reached and kept constant. Each production trajectory was repeated four times from the same starting point to obtain better statistics and insight into the dimerization process. The system sizes were as follows: (1) the benzene dimer system consisted of 9189 atoms, of which 24 belonged to two benzene molecules, and the cell size was 47 Å × 47 Å × 48 Å; (2) the anthracene dimer system consisted of 13,935 atoms, of which 13,887 were water atoms (48 atoms belonged to two anthracene molecules), and the cell size was 53 Å × 51 Å × 58 Å (Figure S4); (3) the phenanthrene dimer system consisted of 11,481 atoms, of which 11,433 were water atoms (48 atoms belonged to two phenanthrene molecules), and the cell size was 47 Å × 53 Å × 52 Å (Figure S5); (4) the R6G dimer system consisted of 21,070 atoms, of which 20,940 were water, and 128 were two R6G molecules, while the last 2 were Cl ions; the cell size was 54 Å × 65 Å × 66 Å (Figure S6). The initial distance between molecules of interest was not smaller than 20 Å, and the water exceeded the system by no less than 20 Å. Because the particular frames of MD simulations were chosen as starting points for SMD simulations, the system sizes and atom count remained unchanged. Initial PAH dimer coordinates are provided in the Supplementary Materials.

In each of the obtained production runs, the most representative stable dimers acquired during the MD trajectories were chosen for the starting configurations of the SMD simulations, resulting in four independent SMD trajectories for each structure of interest. For the SMD simulations, a dummy atom, connected via a virtual spring to the atom that the force was applied to, was introduced. By empirically testing SMD parameters, it was found that the most optimal parameters were a pulling velocity of 0.01 Å/ps and harmonic constraint force constant of 4 kcal/(mol Å) equivalent to 278 pN/Å. In the case of a lower velocity, the simulation timescale became no longer efficient, while the usage of any other force constant resulted in excess noise in the trajectories that dominated over the local unbinding potential.

In all trajectories, one of the dimer components was fixed to reduce the noise, while the other compound was pulled away. To further minimize the noise, constant temperature control was disabled to ensure that the disturbance caused by the molecular movement was minimal. Other parameters remained as in MD simulations. The force plot vs. time and the compound displacement were used to calculate the dissociation energies.

All calculations were performed on 10 CPUs of Nvidia A100 GPU with the CUDA integrator switched off. The average times for SMD simulations were as follows: 29 min for anthracene, 23 min for phenanthrene, and 35 min for R6G. It is worth mentioning that the simulation time can be significantly reduced by using a smaller water box and pre-orienting the molecules. The relatively large systems employed in this work were deliberately chosen to mitigate the possibility of errors that might arise from biasing the system towards unnatural behaviors, particularly in the context of low binding energies, and to ensure the consistency of the results.

Nevertheless, in future works, we would recommend using pre-oriented molecules, a smaller water box, and a 2 fs time step in the MD part and an even smaller system for the SMD simulations.

DFT calculations were performed using ORCA 5.0.4 [42,43] on a single node of ARCHIE-WeSt (192 GB of RAM per node/4.8 GB per core with Intel Xeon Gold 6138 Skylake processors @2.0 GHz with 40 cores per node). A ωB97X-D3 [24] range-separated hybrid functional utilizing the atom-pairwise dispersion correction with the zero-damping scheme introduced by S. Grimme et al. [25] and the cc-pVQZ basis set were used for all calculations, as it was found to give the best balance between the accuracy and computational speed. To obtain the energies of the solvated system, the universal continuum solvation model was employed [44]. Binding energies were obtained using:

(3) $${\Delta E}_{DFT}=E_{monomer\;1}+E_{monomer\;2}-E_{dimer}$$

where $E_{monomer\;1}$ and $E_{monomer\;2}$ are the single point energies of the individual monomers that form the dimer, and $E_{dimer}$ is the single point energy of a dimer. All DFT calculations were performed using the dimer structures used for the SMD trajectories without any prior geometry optimization.

## Figures and Tables

**Table 1:** Exemplar vdW parameters from CHARMM36 FF [22].

Atom Name	ε	Rmin/2	Chemical Group
C	−0.110	2.0000	Pure NMA *
CA	−0.070	1.9924	Benzene
CC	−0.070	2.0000	Acetic Acid
CE1	−0.068	2.0900	Propene
CE2	−0.064	2.0800	Ethene
C3	−0.020	2.2750	Cyclopropane
H	−0.046	0.2245	TIP3P **
HA	−0.022	1.3200	Methane/Ethane
OB	−0.120	1.7000	Acetic Acid Carbonyl
OH1	−0.152	1.7700	TIP3P **

* NMA—*N*-Methylaniline. ** TIP3P—3-site rigid water molecule commonly used in molecular dynamics simulations [23].

**Table 2:** Binding energies obtained from SMD simulations (Δ*E*_SMD_) and DFT-obtained binding energies in gas (${\Delta E}_{DFT}^{Gas}$) and solute (${\Delta E}_{DFT}^{Solvent}$) for different simulated systems (average of four independent repetitions). All values are in kcal/mol.

System	Δ*E*_SMD_	${\Delta E}_{DFT}^{Gas}$	${\Delta E}_{DFT}^{Solvent}$
Anthracene	10.23 ± 1.36	7.40 ± 0.87	9.41 ± 0.64
Phenanthrene	10.34 ± 2.24	9.44 ± 0.74	11.04 ± 0.36
R6G Dimer	8.52 ± 2.80	−19.06 ± 2.16	13.45 ± 3.18

## Data Availability

All data underpinning this publication required to reproduce the simulations are openly available from the University of Strathclyde KnowledgeBase at https://doi.org/10.15129/d244b8e7-8c99-44a2-b595-c51c718694b5 (accessed on 25 May 2024). Any additional data needed will be shared upon reasonable request to the corresponding author.

## Supplementary Materials

The following supporting information can be downloaded at: https://www.mdpi.com/article/10.3390/ijms25115783/s1.
