# Supplementary material for: Estimating Binding Energies of π-Stacked Aromatic Dimers Using Force Field-Driven Molecular Dynamics
Source: Int J Mol Sci. 2024 May 26;25(11):5783. doi: 10.3390/ijms25115783 (PMC11171666; doi:10.3390/ijms25115783)
Supplement: Supplementary file 1 [file ijms-25-05783-s001.zip › Supplementary-KKO_DD.pdf]

How can Classical Molecular Dynamics be used to Estimate the Binding Energies of π-Stacked Aromatic Dimers?

Daniel Doveiko [1], Karina Kubiak-Ossowska [2] and Yu Chen [1,*]

[1] Photophysics Group, Department of Physics, University of Strathclyde, Scottish Universities Physics
Alliance, Glasgow G4 0NG, U.K.
[2] Department of Physics/ARCHIE-WeSt, University of Strathclyde, Glasgow G4 0NG, Scotland
* Correspondence: y.chen@strath.ac.uk

Supplementary Materials

Supplementary materials contain: (1) Supplementary Figures, (2) Coordinates of PAH and R6G Dimers

(1) Supplementary Figures

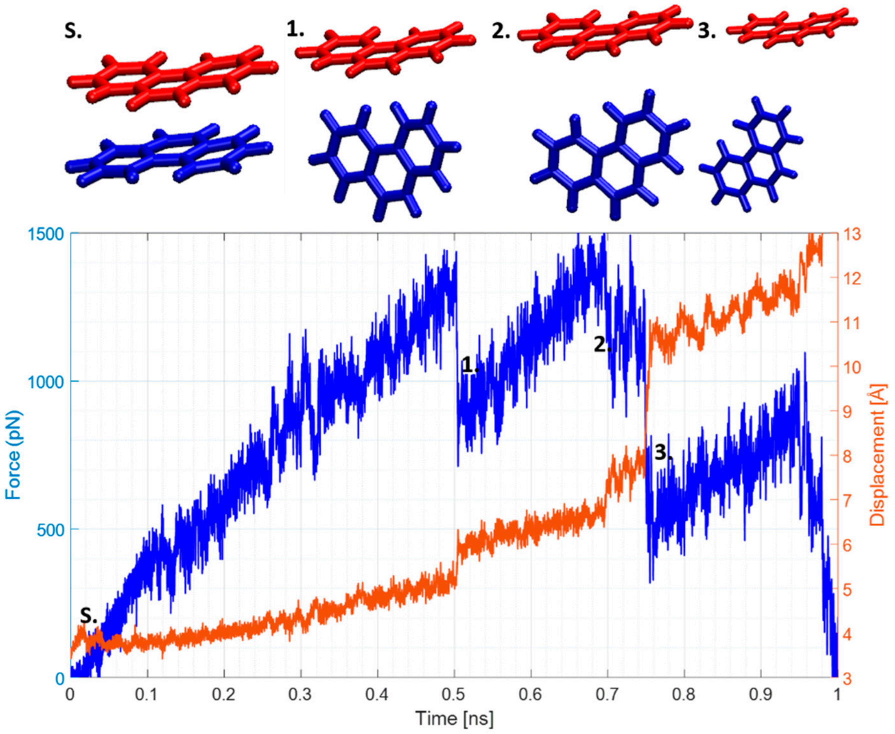

Figure S1. Phenanthrene SMD plots. Structure S represents the starting structures, while structures denoted as 1-3 represent the various stages of dimer dissociation in the SMD trajectory. The blue curve represents the force in pN while the red one the displacement in Å from the original position.

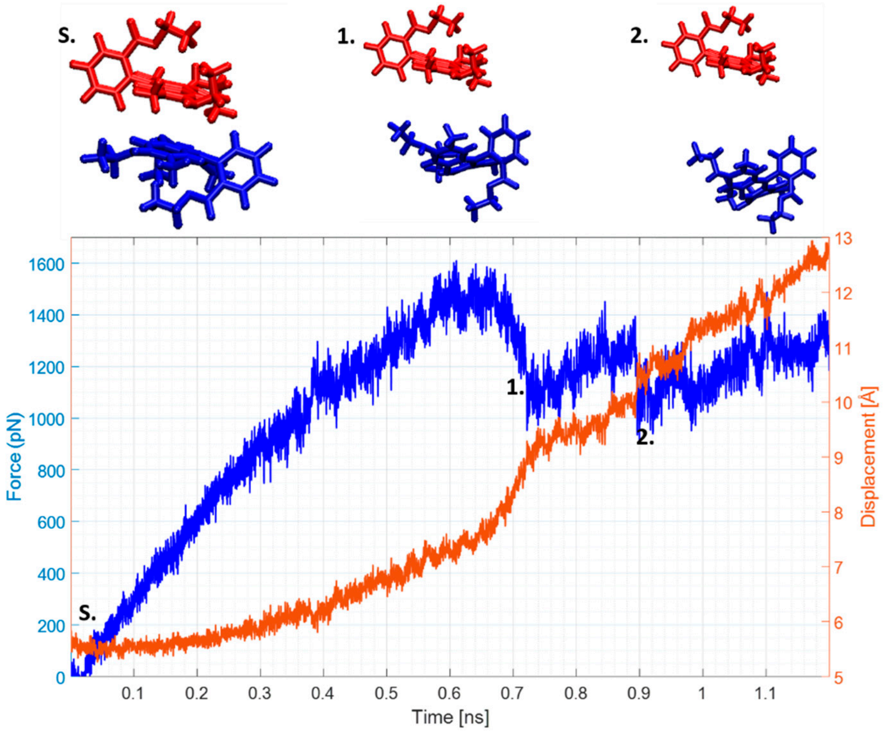

Figure S2. R6G SMD plots. Structure S represents the starting structures, while structures denoted as 1 and 2 represent the various stages of dimer dissociation in the SMD trajectory. Blue curve represents the force in pN while the red one the displacement in Å from original position.

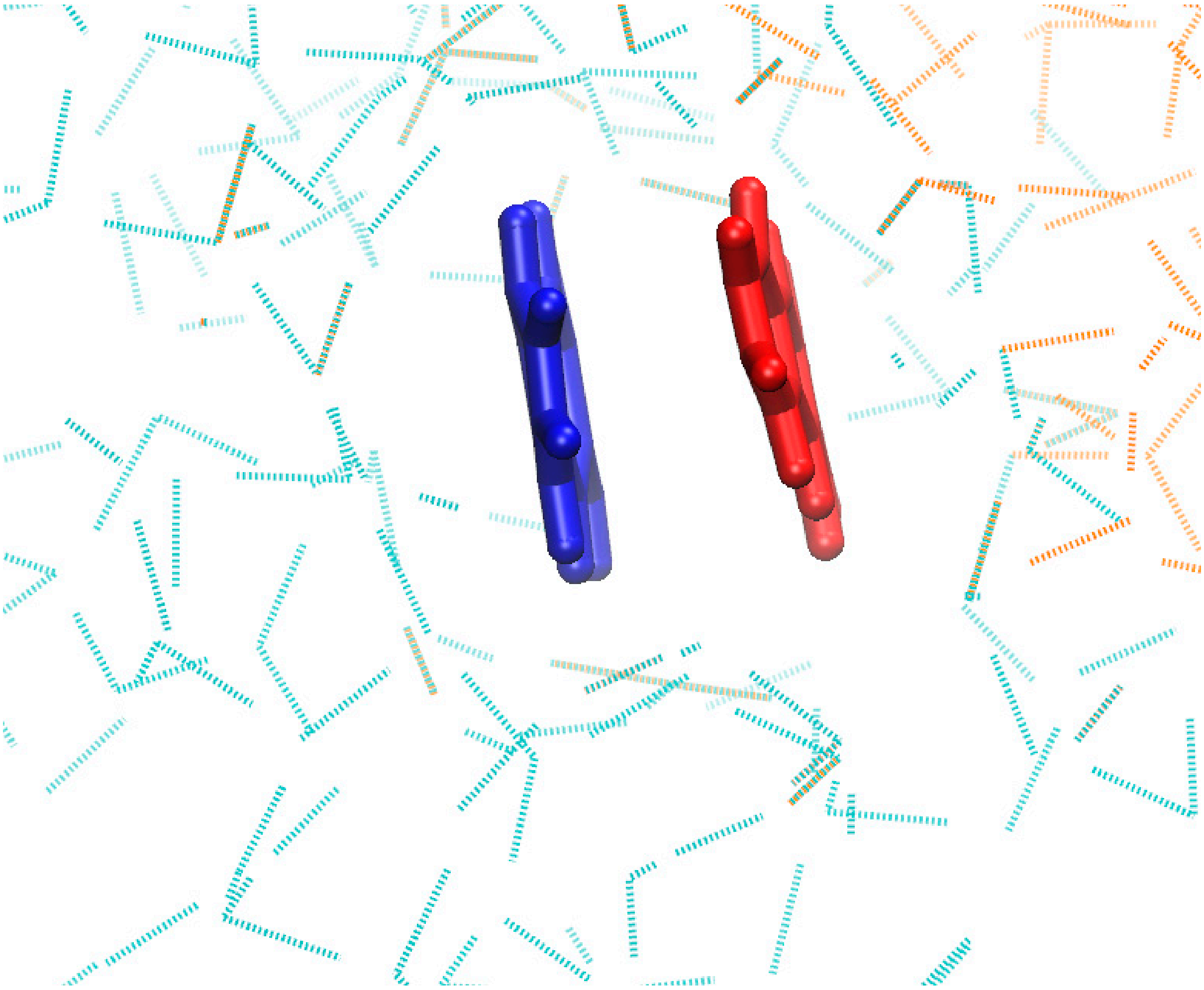

Figure S3. Side view of anthracene molecules in a cavity with no water molecules interacting with them. For clarity water molecules present in the system are shown by H-bond representation and the depth cueing option is used.

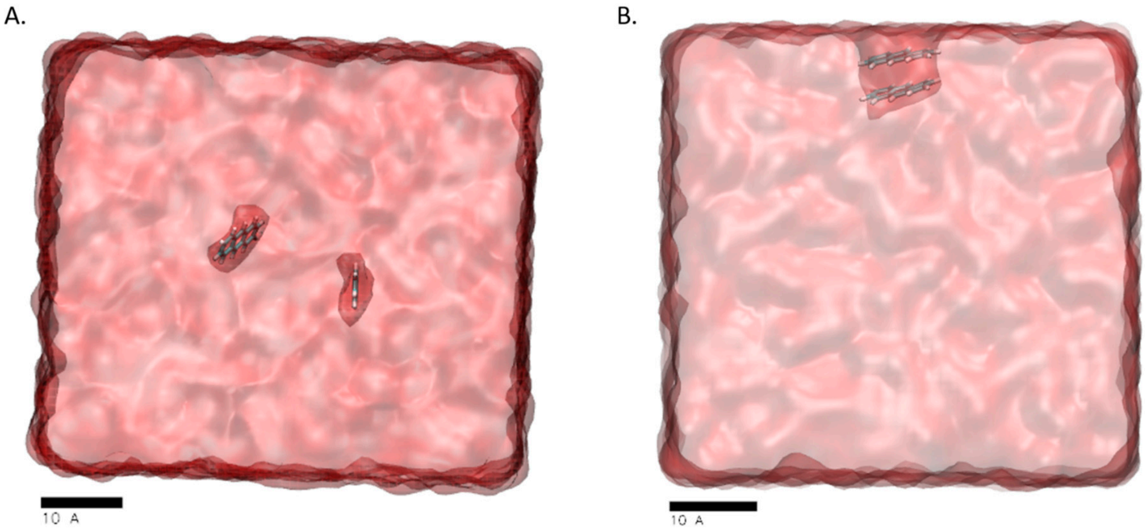

Figure S4. Initial configuration of the anthracene systems. A. MD simulation; B. SMD simulation. The anthracene dimer system consisted of 13,935 atoms of which 13,887 were water atoms (48 atoms belonged to two anthracene molecules) and a cell size was 53 Å × 51 Å × 58 Å.

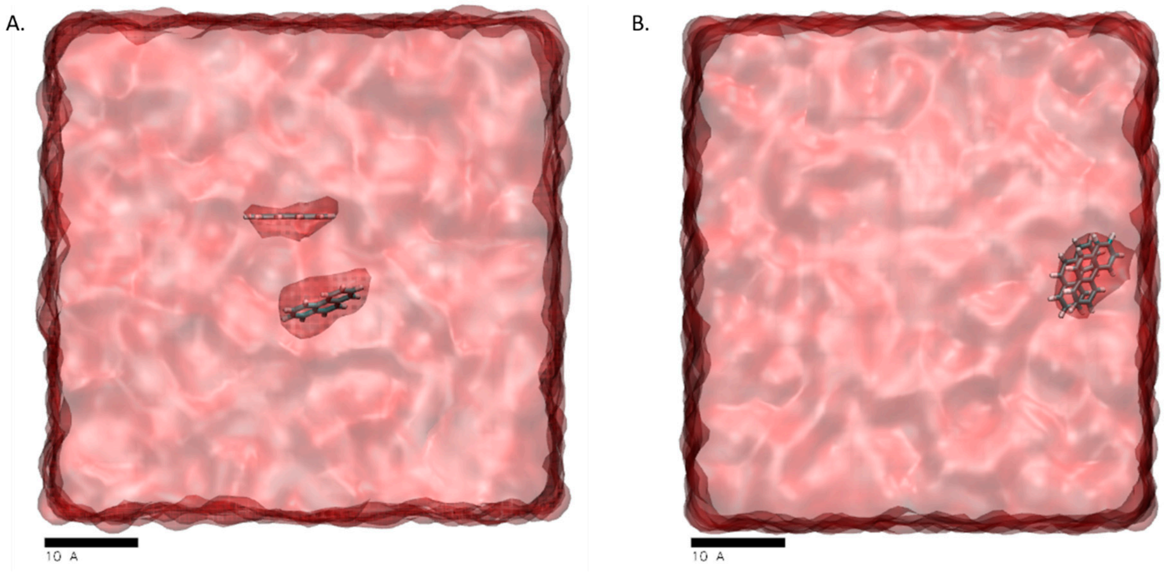

Figure S5. Initial configuration of the phenanthrene systems. A. MD simulation; B. SMD simulation. The phenanthrene dimer system consisted of 11,481 of which 11,433 were water atoms (48 atoms belonged to two phenanthrene molecules) and the cell size was 47 Å × 53 Å × 52 Å.

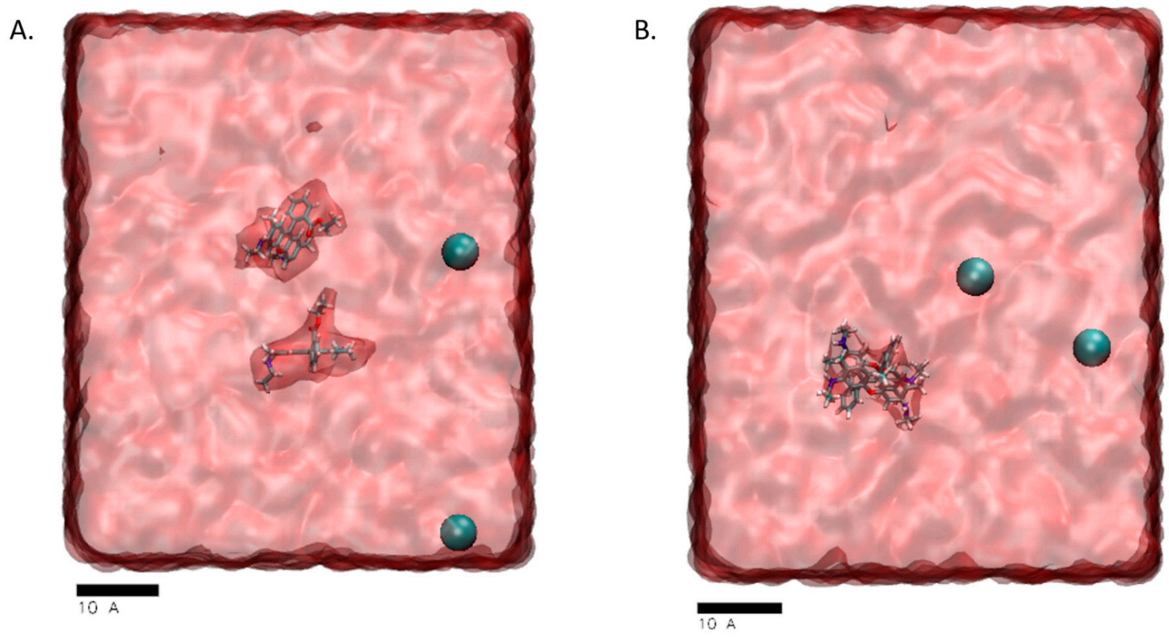

Figure S6. Initial configuration of the R6G systems. A. MD simulation; B. SMD simulation. The R6G dimer system consisted of 21,070 atoms of which 20,940 were water, 128 were two R6G molecules while the last two were Cl ions, and a cell size was 54 Å × 65 Å × 66 Å.

(2) Coordinates of PAH and R6G Dimers

Dimer Coordinates

Anthracene Dimer

ATOM	1	C2	GP00H	1	-2.764	22.383	5.374	1.00	0.00	HAA1	C
ATOM	2	C3	GP00H	1	-1.421	22.778	5.428	1.00	0.00	HAA1	C
ATOM	3	C4	GP00H	1	-0.766	22.686	6.653	1.00	0.00	HAA1	C
ATOM	4	C5	GP00H	1	-1.397	22.185	7.830	1.00	0.00	HAA1	C
ATOM	5	C6	GP00H	1	-2.796	21.857	7.737	1.00	0.00	HAA1	C
ATOM	6	C7	GP00H	1	-3.469	21.958	6.552	1.00	0.00	HAA1	C
ATOM	7	H2	GP00H	1	-3.387	22.403	4.403	1.00	0.00	HAA1	H
ATOM	8	H3	GP00H	1	-1.070	22.967	4.482	1.00	0.00	HAA1	H
ATOM	9	H6	GP00H	1	-3.329	21.382	8.573	1.00	0.00	HAA1	H
ATOM	10	H7	GP00H	1	-4.534	21.768	6.378	1.00	0.00	HAA1	H
ATOM	11	C1	GP00H	2	0.569	23.064	6.699	1.00	0.00	HAA1	C
ATOM	12	C2	GP00H	2	1.318	22.849	7.895	1.00	0.00	HAA1	C
ATOM	13	C3	GP00H	2	2.711	23.124	7.905	1.00	0.00	HAA1	C
ATOM	14	C4	GP00H	2	3.388	23.064	9.136	1.00	0.00	HAA1	C
ATOM	15	C5	GP00H	2	2.670	22.773	10.257	1.00	0.00	HAA1	C
ATOM	16	C6	GP00H	2	1.344	22.428	10.231	1.00	0.00	HAA1	C
ATOM	17	C7	GP00H	2	0.627	22.404	9.022	1.00	0.00	HAA1	C
ATOM	18	C8	GP00H	2	-0.726	22.096	9.073	1.00	0.00	HAA1	C
ATOM	19	H1	GP00H	2	1.087	23.454	5.822	1.00	0.00	HAA1	H
ATOM	20	H3	GP00H	2	3.242	23.338	6.999	1.00	0.00	HAA1	H
ATOM	21	H4	GP00H	2	4.448	23.160	9.327	1.00	0.00	HAA1	H
ATOM	22	H5	GP00H	2	3.282	22.657	11.200	1.00	0.00	HAA1	H
ATOM	23	H6	GP00H	2	0.834	22.163	11.166	1.00	0.00	HAA1	H
ATOM	24	H8	GP00H	2	-1.166	21.811	9.967	1.00	0.00	HAA1	H
ATOM	25	C2	GP00H	1	-3.255	18.117	6.077	1.00	0.00	HAA2	C
ATOM	26	C3	GP00H	1	-2.560	17.816	7.250	1.00	0.00	HAA2	C
ATOM	27	C4	GP00H	1	-1.217	18.205	7.380	1.00	0.00	HAA2	C
ATOM	28	C5	GP00H	1	-0.584	18.838	6.285	1.00	0.00	HAA2	C
ATOM	29	C6	GP00H	1	-1.225	18.870	5.004	1.00	0.00	HAA2	C
ATOM	30	C7	GP00H	1	-2.588	18.556	4.958	1.00	0.00	HAA2	C
ATOM	31	H2	GP00H	1	-4.325	17.743	6.144	1.00	0.00	HAA2	H
ATOM	32	H3	GP00H	1	-3.105	17.366	8.101	1.00	0.00	HAA2	H
ATOM	33	H6	GP00H	1	-0.714	19.268	4.113	1.00	0.00	HAA2	H
ATOM	34	H7	GP00H	1	-3.107	18.694	4.003	1.00	0.00	HAA2	H
ATOM	35	C1	GP00H	2	-0.521	18.139	8.641	1.00	0.00	HAA2	C
ATOM	36	C2	GP00H	2	0.783	18.548	8.721	1.00	0.00	HAA2	C
ATOM	37	C3	GP00H	2	1.439	18.637	9.981	1.00	0.00	HAA2	C
ATOM	38	C4	GP00H	2	2.748	19.135	10.108	1.00	0.00	HAA2	C
ATOM	39	C5	GP00H	2	3.418	19.596	8.990	1.00	0.00	HAA2	C
ATOM	40	C6	GP00H	2	2.824	19.538	7.703	1.00	0.00	HAA2	C
ATOM	41	C7	GP00H	2	1.494	18.990	7.537	1.00	0.00	HAA2	C
ATOM	42	C8	GP00H	2	0.756	19.149	6.361	1.00	0.00	HAA2	C
ATOM	43	H1	GP00H	2	-1.043	17.671	9.509	1.00	0.00	HAA2	H
ATOM	44	H3	GP00H	2	0.959	18.355	10.886	1.00	0.00	HAA2	H

ATOM	45	H4	GP00H	2	3.132	19.054	11.131	1.00	0.00	HAA2	H
ATOM	46	H5	GP00H	2	4.444	19.950	8.981	1.00	0.00	HAA2	H
ATOM	47	H6	GP00H	2	3.355	19.851	6.828	1.00	0.00	HAA2	H
ATOM	48	H8	GP00H	2	1.237	19.574	5.449	1.00	0.00	HAA2	H

Phenanthrene Dimer

ATOM	1	C2	GP00H	1	16.865	2.717	8.554	1.00	0.00	HAA1	C
ATOM	2	C3	GP00H	1	17.890	3.013	7.729	1.00	0.00	HAA1	C
ATOM	3	C4	GP00H	1	18.543	2.004	7.075	1.00	0.00	HAA1	C
ATOM	4	C5	GP00H	1	18.220	0.646	7.307	1.00	0.00	HAA1	C
ATOM	5	C6	GP00H	1	17.151	0.280	8.157	1.00	0.00	HAA1	C
ATOM	6	C7	GP00H	1	16.495	1.386	8.783	1.00	0.00	HAA1	C
ATOM	7	C8	GP00H	1	15.401	1.115	9.560	1.00	0.00	HAA1	C
ATOM	8	H2	GP00H	1	16.387	3.496	9.136	1.00	0.00	HAA1	H
ATOM	9	H3	GP00H	1	18.153	4.052	7.622	1.00	0.00	HAA1	H
ATOM	10	H4	GP00H	1	19.383	2.328	6.429	1.00	0.00	HAA1	H
ATOM	11	H5	GP00H	1	18.754	-0.081	6.705	1.00	0.00	HAA1	H
ATOM	12	H8	GP00H	1	14.889	1.901	10.116	1.00	0.00	HAA1	H
ATOM	13	C1	GP00H	2	14.883	-0.227	9.680	1.00	0.00	HAA1	C
ATOM	14	C2	GP00H	2	15.413	-1.240	8.953	1.00	0.00	HAA1	C
ATOM	15	C3	GP00H	2	16.678	-1.080	8.275	1.00	0.00	HAA1	C
ATOM	16	C4	GP00H	2	17.285	-2.151	7.658	1.00	0.00	HAA1	C
ATOM	17	C5	GP00H	2	16.690	-3.357	7.618	1.00	0.00	HAA1	C
ATOM	18	C6	GP00H	2	15.428	-3.586	8.224	1.00	0.00	HAA1	C
ATOM	19	C7	GP00H	2	14.793	-2.490	8.840	1.00	0.00	HAA1	C
ATOM	20	H1	GP00H	2	14.002	-0.206	10.171	1.00	0.00	HAA1	H
ATOM	21	H4	GP00H	2	18.236	-2.028	7.077	1.00	0.00	HAA1	H
ATOM	22	H5	GP00H	2	17.202	-4.152	7.110	1.00	0.00	HAA1	H
ATOM	23	H6	GP00H	2	14.932	-4.467	8.187	1.00	0.00	HAA1	H
ATOM	24	H7	GP00H	2	13.813	-2.691	9.270	1.00	0.00	HAA1	H
ATOM	25	C2	GP00H	1	16.230	-1.887	12.684	1.00	0.00	HAA2	C
ATOM	26	C3	GP00H	1	16.638	-3.015	11.938	1.00	0.00	HAA2	C
ATOM	27	C4	GP00H	1	17.864	-3.018	11.256	1.00	0.00	HAA2	C
ATOM	28	C5	GP00H	1	18.547	-1.790	11.101	1.00	0.00	HAA2	C
ATOM	29	C6	GP00H	1	18.076	-0.690	11.764	1.00	0.00	HAA2	C
ATOM	30	C7	GP00H	1	16.998	-0.705	12.633	1.00	0.00	HAA2	C
ATOM	31	C8	GP00H	1	16.530	0.479	13.244	1.00	0.00	HAA2	C
ATOM	32	H2	GP00H	1	15.392	-1.916	13.283	1.00	0.00	HAA2	H
ATOM	33	H3	GP00H	1	15.946	-3.892	11.924	1.00	0.00	HAA2	H
ATOM	34	H4	GP00H	1	18.172	-3.964	10.782	1.00	0.00	HAA2	H
ATOM	35	H5	GP00H	1	19.349	-1.718	10.414	1.00	0.00	HAA2	H
ATOM	36	H8	GP00H	1	15.585	0.531	13.785	1.00	0.00	HAA2	H
ATOM	37	C1	GP00H	2	17.124	1.701	12.999	1.00	0.00	HAA2	C
ATOM	38	C2	GP00H	2	18.223	1.792	12.225	1.00	0.00	HAA2	C
ATOM	39	C3	GP00H	2	18.761	0.576	11.618	1.00	0.00	HAA2	C
ATOM	40	C4	GP00H	2	19.968	0.689	10.899	1.00	0.00	HAA2	C
ATOM	41	C5	GP00H	2	20.496	1.895	10.638	1.00	0.00	HAA2	C
ATOM	42	C6	GP00H	2	19.938	3.093	11.138	1.00	0.00	HAA2	C
ATOM	43	C7	GP00H	2	18.818	3.012	11.989	1.00	0.00	HAA2	C
ATOM	44	H1	GP00H	2	16.761	2.498	13.624	1.00	0.00	HAA2	H
ATOM	45	H4	GP00H	2	20.431	-0.078	10.312	1.00	0.00	HAA2	H

ATOM	46	H5	GP00H	2	21.372	1.916	10.000	1.00	0.00	HAA2	H
ATOM	47	H6	GP00H	2	20.428	4.063	10.972	1.00	0.00	HAA2	H
ATOM	48	H7	GP00H	2	18.429	3.907	12.320	1.00	0.00	HAA2	H

R6G Dimer

ATOM	1	C7	R6G	H	21	15.701	-3.855	19.219	1.00	0.00	HAA1	C
ATOM	2	C3	R6G	H	21	14.880	-4.790	19.932	1.00	0.00	HAA1	C
ATOM	3	C2	R6G	H	21	13.697	-5.337	19.346	1.00	0.00	HAA1	C
ATOM	4	C1	R6G	H	21	12.817	-6.198	19.976	1.00	0.00	HAA1	C
ATOM	5	H3	R6G	H	21	11.894	-6.523	19.528	1.00	0.00	HAA1	H
ATOM	6	C6	R6G	H	21	13.155	-6.552	21.314	1.00	0.00	HAA1	C
ATOM	7	N1	R6G	H	21	12.186	-7.320	21.901	1.00	0.00	HAA1	N
ATOM	8	H1	R6G	H	21	12.351	-7.567	22.855	1.00	0.00	HAA1	H
ATOM	9	C25	R6G	H	21	11.704	-8.548	21.259	1.00	0.00	HAA1	C
ATOM	10	C26	R6G	H	21	12.939	-9.394	21.007	1.00	0.00	HAA1	C
ATOM	11	H24	R6G	H	21	11.242	-8.194	20.308	1.00	0.00	HAA1	H
ATOM	12	H23	R6G	H	21	11.036	-9.148	21.900	1.00	0.00	HAA1	H
ATOM	13	H25	R6G	H	21	13.579	-9.426	21.928	1.00	0.00	HAA1	H
ATOM	14	H27	R6G	H	21	13.418	-9.072	19.986	1.00	0.00	HAA1	H
ATOM	15	H26	R6G	H	21	12.632	-10.473	20.817	1.00	0.00	HAA1	H
ATOM	16	C5	R6G	H	21	14.365	-6.125	21.916	1.00	0.00	HAA1	C
ATOM	17	C21	R6G	H	21	14.620	-6.485	23.403	1.00	0.00	HAA1	C
ATOM	18	H16	R6G	H	21	13.771	-6.462	24.034	1.00	0.00	HAA1	H
ATOM	19	H15	R6G	H	21	15.301	-5.711	23.810	1.00	0.00	HAA1	H
ATOM	20	H17	R6G	H	21	15.121	-7.533	23.453	1.00	0.00	HAA1	H
ATOM	21	C4	R6G	H	21	15.309	-5.365	21.181	1.00	0.00	HAA1	C
ATOM	22	H4	R6G	H	21	16.245	-5.063	21.661	1.00	0.00	HAA1	H
ATOM	23	O1	R6G	H	21	13.388	-4.937	18.001	1.00	0.00	HAA1	O
ATOM	24	C9	R6G	H	21	14.295	-4.247	17.308	1.00	0.00	HAA1	C
ATOM	25	C8	R6G	H	21	15.387	-3.538	17.938	1.00	0.00	HAA1	C
ATOM	26	C13	R6G	H	21	16.100	-2.545	17.109	1.00	0.00	HAA1	C
ATOM	27	H7	R6G	H	21	17.026	-2.008	17.537	1.00	0.00	HAA1	H
ATOM	28	C12	R6G	H	21	15.749	-2.191	15.820	1.00	0.00	HAA1	C
ATOM	29	C11	R6G	H	21	14.531	-2.828	15.222	1.00	0.00	HAA1	C
ATOM	30	N2	R6G	H	21	14.608	-2.891	13.728	1.00	0.00	HAA1	N
ATOM	31	C23	R6G	H	21	13.395	-3.225	13.017	1.00	0.00	HAA1	C
ATOM	32	H2	R6G	H	21	15.391	-3.526	13.448	1.00	0.00	HAA1	H
ATOM	33	H18	R6G	H	21	13.092	-4.266	13.298	1.00	0.00	HAA1	H
ATOM	34	H19	R6G	H	21	13.567	-3.123	11.905	1.00	0.00	HAA1	H
ATOM	35	C24	R6G	H	21	12.244	-2.212	13.286	1.00	0.00	HAA1	C
ATOM	36	H21	R6G	H	21	12.022	-2.268	14.400	1.00	0.00	HAA1	H
ATOM	37	H20	R6G	H	21	11.354	-2.259	12.670	1.00	0.00	HAA1	H
ATOM	38	H22	R6G	H	21	12.657	-1.221	13.121	1.00	0.00	HAA1	H
ATOM	39	C10	R6G	H	21	13.992	-4.113	15.854	1.00	0.00	HAA1	C
ATOM	40	H5	R6G	H	21	12.937	-4.511	15.562	1.00	0.00	HAA1	H
ATOM	41	C20	R6G	H	21	16.509	-1.046	15.165	1.00	0.00	HAA1	C
ATOM	42	H12	R6G	H	21	16.817	-1.384	14.155	1.00	0.00	HAA1	H
ATOM	43	H13	R6G	H	21	17.363	-0.730	15.756	1.00	0.00	HAA1	H
ATOM	44	H14	R6G	H	21	15.785	-0.170	14.986	1.00	0.00	HAA1	H

ATOM	45	C14	R6G	H	21	17.032	-3.372	19.798	1.00	0.00	HAA1 C
ATOM	46	C19	R6G	H	21	18.274	-3.952	19.557	1.00	0.00	HAA1 C
ATOM	47	C22	R6G	H	21	18.497	-5.353	18.962	1.00	0.00	HAA1 C
ATOM	48	O2	R6G	H	21	17.395	-5.940	18.631	1.00	0.00	HAA1 O
ATOM	49	O3	R6G	H	21	19.600	-5.905	18.876	1.00	0.00	HAA1 O
ATOM	50	C27	R6G	H	21	17.660	-7.329	18.277	1.00	0.00	HAA1 C
ATOM	51	C28	R6G	H	21	16.312	-7.959	18.112	1.00	0.00	HAA1 C
ATOM	52	H29	R6G	H	21	18.246	-7.820	19.068	1.00	0.00	HAA1 H
ATOM	53	H28	R6G	H	21	18.207	-7.399	17.299	1.00	0.00	HAA1 H
ATOM	54	H32	R6G	H	21	15.662	-7.826	19.019	1.00	0.00	HAA1 H
ATOM	55	H30	R6G	H	21	16.438	-8.966	17.891	1.00	0.00	HAA1 H
ATOM	56	H31	R6G	H	21	15.860	-7.580	17.217	1.00	0.00	HAA1 H
ATOM	57	C18	R6G	H	21	19.444	-3.372	20.021	1.00	0.00	HAA1 C
ATOM	58	C17	R6G	H	21	19.448	-2.205	20.791	1.00	0.00	HAA1 C
ATOM	59	H11	R6G	H	21	20.380	-3.883	19.824	1.00	0.00	HAA1 H
ATOM	60	H10	R6G	H	21	20.408	-1.743	21.110	1.00	0.00	HAA1 H
ATOM	61	C16	R6G	H	21	18.132	-1.714	21.192	1.00	0.00	HAA1 C
ATOM	62	C15	R6G	H	21	16.996	-2.274	20.671	1.00	0.00	HAA1 C
ATOM	63	H9	R6G	H	21	17.962	-0.895	21.820	1.00	0.00	HAA1 H
ATOM	64	H8	R6G	H	21	16.062	-1.747	21.044	1.00	0.00	HAA1 H
ATOM	65	C7	R6G	H	21	10.043	-3.124	19.688	1.00	0.00	HAA2 C
ATOM	66	C3	R6G	H	21	10.822	-3.393	20.891	1.00	0.00	HAA2 C
ATOM	67	C2	R6G	H	21	12.119	-2.855	20.996	1.00	0.00	HAA2 C
ATOM	68	C1	R6G	H	21	12.816	-2.909	22.219	1.00	0.00	HAA2 C
ATOM	69	H3	R6G	H	21	13.798	-2.447	22.298	1.00	0.00	HAA2 H
ATOM	70	C6	R6G	H	21	12.280	-3.536	23.358	1.00	0.00	HAA2 C
ATOM	71	N1	R6G	H	21	12.989	-3.311	24.537	1.00	0.00	HAA2 N
ATOM	72	H1	R6G	H	21	12.705	-3.913	25.350	1.00	0.00	HAA2 H
ATOM	73	C25	R6G	H	21	13.021	-1.841	24.884	1.00	0.00	HAA2 C
ATOM	74	C26	R6G	H	21	13.531	-1.702	26.339	1.00	0.00	HAA2 C
ATOM	75	H24	R6G	H	21	11.957	-1.488	24.831	1.00	0.00	HAA2 H
ATOM	76	H23	R6G	H	21	13.610	-1.311	24.130	1.00	0.00	HAA2 H
ATOM	77	H25	R6G	H	21	13.760	-0.693	26.583	1.00	0.00	HAA2 H
ATOM	78	H27	R6G	H	21	14.430	-2.349	26.590	1.00	0.00	HAA2 H
ATOM	79	H26	R6G	H	21	12.799	-2.066	27.038	1.00	0.00	HAA2 H
ATOM	80	C5	R6G	H	21	11.014	-4.252	23.197	1.00	0.00	HAA2 C
ATOM	81	C21	R6G	H	21	10.138	-4.729	24.397	1.00	0.00	HAA2 C
ATOM	82	H16	R6G	H	21	9.559	-3.913	24.741	1.00	0.00	HAA2 H
ATOM	83	H15	R6G	H	21	10.824	-4.963	25.251	1.00	0.00	HAA2 H
ATOM	84	H17	R6G	H	21	9.541	-5.623	24.207	1.00	0.00	HAA2 H
ATOM	85	C4	R6G	H	21	10.332	-4.122	21.964	1.00	0.00	HAA2 C
ATOM	86	H4	R6G	H	21	9.335	-4.607	22.009	1.00	0.00	HAA2 H
ATOM	87	O1	R6G	H	21	12.691	-2.212	19.981	1.00	0.00	HAA2 O
ATOM	88	C9	R6G	H	21	11.850	-1.646	19.066	1.00	0.00	HAA2 C
ATOM	89	C8	R6G	H	21	10.552	-2.135	18.773	1.00	0.00	HAA2 C
ATOM	90	C13	R6G	H	21	9.786	-1.746	17.494	1.00	0.00	HAA2 C
ATOM	91	H7	R6G	H	21	8.753	-1.973	17.328	1.00	0.00	HAA2 H
ATOM	92	C12	R6G	H	21	10.420	-0.930	16.648	1.00	0.00	HAA2 C
ATOM	93	C11	R6G	H	21	11.741	-0.140	17.015	1.00	0.00	HAA2 C
ATOM	94	N2	R6G	H	21	12.544	0.424	15.857	1.00	0.00	HAA2 N
ATOM	95	C23	R6G	H	21	13.096	1.781	16.095	1.00	0.00	HAA2 C

ATOM	96	H2	R6G	H	21	12.031	0.418	14.953	1.00	0.00	HAA2	H
ATOM	97	H18	R6G	H	21	13.694	2.047	15.164	1.00	0.00	HAA2	H
ATOM	98	H19	R6G	H	21	13.734	1.731	17.006	1.00	0.00	HAA2	H
ATOM	99	C24	R6G	H	21	11.999	2.867	16.281	1.00	0.00	HAA2	C
ATOM	100	H21	R6G	H	21	12.490	3.778	16.642	1.00	0.00	HAA2	H
ATOM	101	H20	R6G	H	21	11.062	2.585	16.900	1.00	0.00	HAA2	H
ATOM	102	H22	R6G	H	21	11.617	3.118	15.293	1.00	0.00	HAA2	H
ATOM	103	C10	R6G	H	21	12.547	-0.630	18.193	1.00	0.00	HAA2	C
ATOM	104	H5	R6G	H	21	13.338	0.019	18.612	1.00	0.00	HAA2	H
ATOM	105	C20	R6G	H	21	9.581	-0.470	15.479	1.00	0.00	HAA2	C
ATOM	106	H12	R6G	H	21	10.298	-0.533	14.596	1.00	0.00	HAA2	H
ATOM	107	H13	R6G	H	21	9.254	0.594	15.506	1.00	0.00	HAA2	H
ATOM	108	H14	R6G	H	21	8.677	-1.176	15.222	1.00	0.00	HAA2	H
ATOM	109	C14	R6G	H	21	8.622	-3.681	19.489	1.00	0.00	HAA2	C
ATOM	110	C19	R6G	H	21	7.449	-3.233	20.120	1.00	0.00	HAA2	C
ATOM	111	C22	R6G	H	21	7.332	-2.251	21.234	1.00	0.00	HAA2	C
ATOM	112	O2	R6G	H	21	8.430	-1.594	21.457	1.00	0.00	HAA2	O
ATOM	113	O3	R6G	H	21	6.268	-2.054	21.806	1.00	0.00	HAA2	O
ATOM	114	C27	R6G	H	21	8.377	-0.765	22.566	1.00	0.00	HAA2	C
ATOM	115	C28	R6G	H	21	9.781	-0.543	23.171	1.00	0.00	HAA2	C
ATOM	116	H29	R6G	H	21	7.792	-1.202	23.382	1.00	0.00	HAA2	H
ATOM	117	H28	R6G	H	21	7.958	0.239	22.224	1.00	0.00	HAA2	H
ATOM	118	H32	R6G	H	21	9.656	-0.274	24.255	1.00	0.00	HAA2	H
ATOM	119	H30	R6G	H	21	10.371	0.095	22.531	1.00	0.00	HAA2	H
ATOM	120	H31	R6G	H	21	10.189	-1.507	23.224	1.00	0.00	HAA2	H
ATOM	121	C18	R6G	H	21	6.253	-3.989	19.858	1.00	0.00	HAA2	C
ATOM	122	C17	R6G	H	21	6.243	-5.172	19.104	1.00	0.00	HAA2	C
ATOM	123	H11	R6G	H	21	5.351	-3.558	20.281	1.00	0.00	HAA2	H
ATOM	124	H10	R6G	H	21	5.355	-5.742	18.848	1.00	0.00	HAA2	H
ATOM	125	C16	R6G	H	21	7.453	-5.625	18.516	1.00	0.00	HAA2	C
ATOM	126	C15	R6G	H	21	8.580	-4.819	18.626	1.00	0.00	HAA2	C
ATOM	127	H9	R6G	H	21	7.544	-6.548	18.031	1.00	0.00	HAA2	H
ATOM	128	H8	R6G	H	21	9.442	-5.042	18.108	1.00	0.00	HAA2	H
